# Assessment of Anatomical Dentin Thickness in Mandibular First Molar: An In Vivo Cone-Beam Computed Tomographic Study

**DOI:** 10.1155/2024/8823070

**Published:** 2024-05-30

**Authors:** Sahil Choudhari, Kavalipurapu Venkata Teja, Sindhu Ramesh, Jerry Jose, Mariangela Cernera, Parisa Soltani, Emmanuel João Nogueira Leal da Silva, Gianrico Spagnuolo

**Affiliations:** ^1^ Department of Conservative Dentistry and Endodontics Saveetha Dental College Saveetha Institute of Medical and Technical Sciences Saveetha University, Chennai, Tamil Nadu, India; ^2^ Private Practice, Kochi, Kerala, India; ^3^ Department of Neuroscience Reproductive and Odontostomatological Sciences University of Naples, Federico II, Via Pansini n°5, Naples 80131, Italy; ^4^ Department of Endodontics School of Dentistry Grande Rio University (UNIGRANRIO), Duque de Caxias, Brazil; ^5^ Department of Endodontics School of Dentistry Rio de Janeiro State University (UERJ), Rio de Janeiro, Brazil

## Abstract

**Aim:**

To determine the minimum dentin thickness in the mesial and distal walls of the mesiobuccal (MB) and mesiolingual (ML) canals of the mandibular first molars using cone-beam computed tomography (CBCT).

**Materials and Methods:**

CBCT examinations of 624 mandibular first molars from an Indian subpopulation were analyzed. The mesial and distal minimum dentin thickness was evaluated in 1 mm intervals apical to the furcation area. Independent *t*-test was used to analyze the data (*α* = 0.05). Using Cohen's kappa coefficient, the interexaminer and intraexaminer reliability was evaluated.

**Results:**

The mesial dentin thickness was significantly higher than the distal dentin thickness for MB and ML canals (*P*=0.01). The average dentin thickness in the distal and mesial plane of the MB canal was 1.15 ± 0.15 mm and 1.52 ± 0.19 mm at the 1 mm level and 0.83 ± 0.13 and 1.08 ± 0.18 at the 5 mm level, respectively. For the ML canal, the average dentin thickness in the distal plane and the mesial plane was 1.24 ± 0.18 mm and 1.44 ± 0.21 at the 1 mm level and 0.91 ± 0.16 and 1.01 ± 0.17 at the 5 mm level, respectively. Statistical analysis between the MB and ML canals showed significant differences in the dentin thickness at 4 and 5 mm levels in both the distal and the mesial planes (*P*=0.01). In more than 85% of the cases, the minimum dentin thickness was seen at the 5 mm level in both the distal and mesial planes in MB and ML canals.

**Conclusion:**

The distal planes of the mesiolingual and mesiobuccal canals were thinner in most cases, making the distal surface more prone to iatrogenic perforations. Considerably, at 4 and 5 mm from the furcation, the distal wall was significantly thinner than the mesial walls. Understanding the anatomy of the danger zone in the mesial roots of the mandibular first molars may serve to minimize the risk of endodontic mishaps such as strip perforations.

## 1. Introduction

Endodontic treatment requires a thorough understanding of the tooth's internal anatomy and morphology, as it allows for efficient root canal preparation and filling [1]. In multiple published reports, it was seen that mandibular first molars are considered to be the most common endodontically treated teeth [2, 3]. In most mandibular molars, approximately 4–7 mm below the furcation, the mesial root has a greater concavity which is generally associated with limited dentin thickness [4]. Hence, preserving the dentin by minimal shaping can have a more favorable long-term prognostic outcome for the tooth [5].

Shaping the root canal using engine-driven instruments can damage the dentinal walls of the root canal, leading to potential iatrogenic errors which can ultimately influence the outcome of the treatment [6, 7, 8]. Since it has been seen that perforations are most commonly seen in the distal area of the mandibular molar mesial roots, Abou-Rass et al. [9] defined the “danger zone” as the portion of the mesial root with a thinner dentin layer in 1980, while the “safety zone” is defined as the mesial portion of the mesial root with a thicker dentin layer. De-Deus et al. [4] have, however, reconsidered the anatomical danger zone concept based on microcomputed tomography (micro-CT) and found that in approximately 40% of canals, the dentin thickness was the smallest on the mesial plane in mandibular molars. One of the important aspects of the application of new engine-driven endodontic systems is their safety in use in the danger zone. If a rotary system removes an excessive amount of dentin from the danger zone, it may lead to the weakening of the root structure or root perforation [7].

Although several approaches have been used to study dentin thickness, micro-CT and cone-beam computed tomography (CBCT) are nondestructive 3D imaging modalities that have been widely used in the last decade [4, 10]. Micro-CT imaging has an advantage due to its superior resolution to perform morphometric measurements [11]. As of now, micro-CT cannot be used for clinical assessment due to its disadvantages, such as its long scanning time, high radiation dose, and size limitations. These constraints make micro-CT inappropriate for assessing a large sample population in contrast to CBCT imaging [12, 13, 14]. Based on the available data and clinical applicability, the use of CBCT is justified in endodontics by the European Society of Endodontology currently recommending it as a standard imaging tool in addition to conventional periapical radiography. CBCT can be used as an appropriate tool for clinical diagnosis, treatment planning, and follow-up of endodontic cases [15, 16]. Additionally, our search revealed limited data on CBCT-based assessments of the minimum dentin thickness in mandibular molars [17, 18]. Although micro-CT provides images with higher resolution than CBCT, it is not possible to use it for in vivo examinations of large samples or with a high and representative sample size, in contrast to CBCT which allows us to obtain the data from the larger sample size [19, 20, 21, 22]. Hence, the present study focused on assessing the minimum dentin thickness in the mesial and distal planes of the mesial roots of mandibular first molars using CBCT as an imaging tool.

## 2. Materials and Methods

### 2.1. Protocol Registration and Sample Size Analysis

This study followed the principles of the Declaration of Helsinki, and the study protocol was approved by the University Ethics Committee (registration number IHEC/SDC/ENDO-2102/21/300). Written informed consent was obtained from all patients before collecting the data. The data were randomly collected from the patient database from 10 January 2021 to 12 December 2021. Power calculation was performed using G^*∗*^Power 3.1 software for Windows (Henrick Heine-Universität, Düsseldorf, Germany) keeping an effect size of 0.1, alpha error of 0.05, and beta error of 0.95 based on the results of a pilot study. A minimum sample size of 486 was determined.

### 2.2. Data Acquisition

The participants' mandibular arches were scanned by an experienced radiologist with 5 years of experience. CBCT scans were obtained from the patients in a standing position using CS 9600 CBCT Scanner (Carestream Dental, Atlanta, GA) at 120 kVp, 4 mA, and 5.5–40 s scan time, with a voxel size of 0.15 mm and field of view of 8 cm × 5 cm following the manufacturer's recommended protocol. The participants were selected according to the following inclusion criteria: CBCT images acquired for endodontic, orthodontic, or implant purposes or diagnosis of impacted teeth or trauma limited to the mandibular molar region. The exclusion criteria included teeth with root resorption, immature apices, signs of fractures, calcifications, presence of dental caries, periapical lesions, odontogenic or nonodontogenic pathology, endodontic treatments, posts, or crowns, as well as artifacts from adjacent implants or metallic restorations.

### 2.3. Radiographic Analysis and Measurements

The acquired images were analyzed in the dedicated Image Viewer (Carestream Dental, Atlanta, GA, USA). The CBCT scans were assessed independently by two endodontists (S.C. and J.J.) with 3 years of experience in evaluating CBCT images on a 28-inch monitor (LU28E590DS, Samsung Electronics, Seoul, Korea) with a pixel resolution of 3,840 × 2,160. The viewing condition for both observers was similar. In order to achieve subjective optimal visualization, the images were adjusted for contrast, brightness, and sharpness. Before evaluation, each evaluator randomly assessed a series of CBCT images that were not associated with this study for calibration. In case of disagreement, a third and fourth endodontist (K.V.T. and E.J.N.L.) with 5 or more years of experience were consulted for consensus.

**Figure 1:**
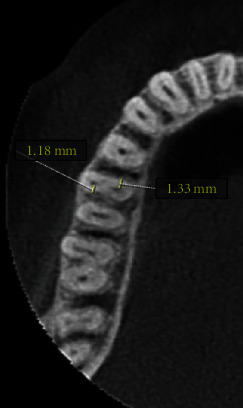
Measurement on the distal wall of the mesiobuccal and the mesial wall of the mesiolingual canals in a right mandibular first molar on axial CBCT image.

Each acquired CBCT image was evaluated in axial sections. The thickness of the root dentin structure (distal and mesial walls) of the mesiobuccal (MB) and mesiolingual (ML) canals was evaluated in the axial section starting from the furcation at five 1 mm intervals apical to the furcation based on a previously published methodology [18]. The examination was conducted by tracing a line starting from the inner wall of the canal and moving perpendicularly to the outer wall of the root. The shortest distance between the canal radiolucency until the external portion of root radiopacity indicates the root dentin (Figure 1). All measurements were performed at 4x magnification, and the measurements were repeated three times to record the mean thickness.

### 2.4. Statistical Analysis

The obtained mean thickness values at five different levels were statistically analyzed using SPSS software version 23.0 (IBM Corporation, Armonk, USA). In addition to descriptive statistics (mean ± SD), the Shapiro–Wilk test was used to analyze adherence to the normal distribution. Between the MB and ML canals, the mean dentin thickness values were compared at five different levels using an independent *t*-test on both mesial and distal planes. *P*  < 0.05 was considered statistically significant. Using Cohen's kappa coefficient, interexaminer and intraexaminer reliability was evaluated.

## 3. Results

CBCT images of 624 mandibular first molars from 381 individuals were studied. There were significant differences in the minimum dentin thickness in the MB canal and ML canal of the mandibular first molars in the distal and mesial planes (Tables 1 and 2).

The selected age group ranged from 18 to 40 years with a mean age of 24.07 ± 10.69. The final images were taken from 59.2% male and 40.8% female patients. The kappa values for the intraexaminer and interexaminer agreement were 0.89 (*P*=0.92) and 0.92 (*P*=0.84), respectively.

Statistical analysis between the MB and ML canal showed significant differences in the dentin thickness at 4 and 5 mm levels in the distal and the mesial planes (*P*=0.01) (Table 1). Descriptive data of the dentin thickness of the distal plane and the mesial plane of the MB and ML canals showed higher values of the dentin thickness in the mesial plane (Table 2). The minimum dentin thickness in the mesial and distal planes of both MB and ML canals was mostly located at the 5 mm level (Table 3). The minimum dentin thickness was toward the mesial plane of the roots in 36% of the canals and the distal plane of the roots in 64% of the canals.

## 4. Discussion

Based on the findings of the present study, the dentin thickness gradually decreased from the furcation toward the apical direction. The mean dentin thickness was higher in the mesial plane compared to the distal plane in both the ML and MB canals of the mesial root. In 36% of canals, dentin thickness was the smallest toward the mesial plane of the roots, while in 64% of canals, it was the smallest toward the distal plane of the roots.

In the present study, through CBCT imaging, the mesial roots of the mandibular molars were evaluated in vivo for root dentin thickness. Generally, the mandibular first molars exhibit more complexities in root canal configurations, limiting the effectiveness of endodontic treatment [8]. The study found that in 64% of the canals, the minimum dentin thickness was in the distal plane of the roots. The results are consistent with prior published reports suggesting that iatrogenic errors such as strip perforation are more prone to occur in the distal walls of the mesial root, the danger zone [23]. Critical knowledge of the root canal system is mandatory for clinicians before the start of the procedure to prevent such types of procedural errors [24]. Additionally, excessive flaring of canals and shaping protocol employed for root canal preparation can further increase the incidence of these errors [25, 26].

The assessed CBCT images of the current study showed that in most cases, the minimum dentin thickness for the MB and ML canals was seen at the 5 mm level in both the distal and mesial planes. These findings are in line with the previously published studies. A study using micro-CT by De-Deus et al. [4] reported that the danger zone level in the distal plane of the mesial roots was toward the middle third of the root (4–7 mm from furcation), supporting the findings of the present study. Furthermore, the minimal dentin thickness of most of the studies ranged from slightly less than 1–3 mm [23, 27, 28]. According to Lim and Stock [28], the remaining dentin thickness has a role in withstanding forces exerted during the root canal treatment procedure. Hence, they advocated that the smallest dentin thickness should be more than 200–300 *µ*m, since thinner dentinal walls can lead to perforation of the root. This further emphasizes the importance of maintaining the dentinal thickness for the long-term prognosis of the tooth [29, 30].

Another finding of this study was that at 4 and 5 mm distances, the minimal dentin thickness in the mesial and distal planes of both the mesiobuccal and mesiolingual roots were statistically different from other levels. This can be a result of the tapering and curvature of the roots, making the dentinal walls significantly smaller in 4 and 5 mm distances from the furcation [18].

The population-based difference influences the minimum dentin thickness. The present study included images from an Indian subpopulation. In a Chinese population, the minimal distal dentin thicknesses of the MB and ML canals were located at 3–4 mm below the furcation for both men and women. Furthermore, the smallest dentin thickness in the distal aspect increased with age irrespective of sex for both MB and ML canals [18]. These findings could be justified by the physiological alterations of the normal dentin to sclerotic dentin with progress in age causing mineralization and ultimately causing narrowing of the tubules [31].

The advent of micro-CT imaging has overcome a plethora of disadvantages in comparison to CBCT showing better accuracy and detail in the assessment of the root canal anatomies of the tooth [32]. While it has been reported to be used on experimental small animal models, the general clinical application of micro-CT is still limited [33].

The findings of the current study are crucial as they will help clinicians prevent errors during root canal shaping and postspace preparations. One of the limitations of this study was selecting a subpopulation for analysis which could have influenced the results of the study. Furthermore, the age and sex of the participants were not taken into consideration. However, the large sample size was one of the strengths of the current study.

## 5. Conclusion

The distal planes of the mesiolingual and mesiobuccal canals were thinner in most cases, making the distal surface more prone to iatrogenic perforations. Considerably, at 4 and 5 mm from the furcation, the distal wall was significantly thinner than the mesial walls. Understanding the anatomy of the danger zone in the mesial roots of mandibular first molars may serve to minimize the risk of endodontic mishaps such as strip perforations.

## Figures and Tables

**Table 1:** Mean ± standard deviation of the dentin thickness of distal plane and mesial plane of the mesiobuccal and mesiolingual canals of the lower first molar at five different levels.

Unit (mm)	Mesiobuccal canal (mm)		Mesiolingual canal (mm)	
	Distal plane	Mesial plane	Distal plane	Mesial plane
1	1.15 ± 0.15^A^	1.52 ± 0.19^A^	1.24 ± 0.18^A^	1.44 ± 0.21^A^
2	1.03 ± 0.15^A^	1.42 ± 0.11^A^	1.11 ± 0.17^A^	1.34 ± 0.19^A^
3	0.95 ± 0.15^A^	1.33 ± 0.13^A^	1.03 ± 0.16^A^	1.26 ± 0.22^A^
4	0.88 ± 0.15^A^	1.19 ± 0.19^B^	1.01 ± 0.18^A^	1.05 ± 0.17^B^
5	0.83 ± 0.13^A^	1.08 ± 0.18^B^	0.91 ± 0.16^A^	1.01 ± 0.17^B^

Different superscript letters represent statistical differences for the same canal and level but different planes (*P*  < 0.05).

**Table 2:** Descriptive data of the dentin thickness (mm) of distal plane and mesial plane of the mesiobuccal and mesiolingual canals of the lower first molar.

	Distal plane of MB canal	Mesial plane of MB canal	Distal plane of ML canal	Mesial plane of ML canal
Mean ± SD	0.975 ± 0.35	1.31 ± 0.42	1.08 ± 0.21	1.20 ± 0.42
Range	0.7–1.5	0.8–2	0.6–1.8	0.6–1.9

**Table 3:** Distribution of the smallest dentin thickness along the cross-sections for all the specimens according to the distance from the furcation area.

Location of the smallest dentin thickness (mm)	Mesiobuccal canal		Mesiolingual canal	
	Distal plane (%)	Mesial plane (%)	Distal plane (%)	Mesial plane (%)
1	0.1	0.8	0.3	1
2	0.3	1.7	0.9	1.3
3	4.1	4.3	4.7	3.4
4	6.1	7.7	6.3	6
5	89.4	85.5	87.8	88.3

## Data Availability

The datasets analyzed during this study are not publicly available but are available from the corresponding author on reasonable request.

## Abbreviations

MB: Mesiobuccal
ML: Mesiolingual
CBCT: Cone beam computed tomography
CT: Computed tomography.
